# Extraction of Intrapulmonary Projectiles

**Published:** 1918-10

**Authors:** 


**On the Extraction of Intrapulmonary Projectiles.** By G. MARION, *Bulletin de la Société de Chirurgie*, August 6, 1918.

Out of 158 patients, Marion treated only 2 cases which he could not operate, and out of the 156 cases operated there was only 1 death. In 7 of these Marion made two attempts to extract the projectile, and in 12 cases there was purulent pleurisy, which, however, was easily cured after incision. His statistics are remarkably good and he attributes them entirely to his special technic. He objects to Petit de la Villéon's technic, which, he admits, has given excellent results, on the ground that it requires operating in the dark.

Duval's method has not given Marion as good results as his own, perhaps for lack of familiarity with it.

Le Fort's technic seems to him to require too great virtuosity, and to yield no results superior to his own. Marion's process is to locate the projectile exactly, and then to resect a rib giving access to it. He fixes the lung to the wall with three or four stitches of catgut, and then, guided by the localizing compass, he makes an incision through the pleural wall between the stitches. When the wall is opened, the projectile is found and removed by the fingers. Only seven times out of 156 cases did he fail to obtain the projectile the first time.

